# Estimating the Economic and Clinical Value of Reducing Antimicrobial Resistance to Three Gram-negative Pathogens in Japan

**DOI:** 10.36469/001c.28327

**Published:** 2021-10-06

**Authors:** Tetsuya Matsumoto, Oliver Darlington, Ryan Miller, Jason Gordon, Phil McEwan, Takahisa Ohashi, Amer Taie, Akira Yuasa

**Affiliations:** 1 International University of Health and Welfare Faculty of Medicine Graduate School of Medicine, Narita, Japan; 2 Health Economics and Outcomes Research Ltd, Cardiff, UK; 3 Pfizer Japan Inc., Tokyo, Japan; 4 Pfizer R&D UK Limited, Tadworth, UK

**Keywords:** anti-microbial resistance, AMR, Japan, Gram-negative, economic burden, burden of illness

## Abstract

**Background:** Antimicrobial resistance (AMR) represents a significant global public health crisis. Despite ample availability of Gram-positive antibiotics, there is a distinct lack of agents against Gram-negative pathogens, including carbapenem-resistant Enterobacterales, which remains a real threat in Japan. The AMR Action Plans aim to mitigate the growing public health concern posed by AMR.

**Objective:** This study aims to estimate the clinical and economic outcomes of drug-resistant Gram-negative pathogens forecasts for Japan to guide resource allocation defined within the upcoming National AMR Action Plan.

**Methods:** A previously published and validated dynamic health economic model was adapted to the Japanese setting. The model used a 10-year time horizon with a willingness-to-pay threshold of ¥5 000 000 (US $46 827) and discounting was applied at a rate of 2% to costs and benefits. Clinical and economic outcomes were assessed as a function of varying AMR levels of three Gram-negative pathogens in Japan by up to 100% of the current level.

**Results:** Reducing drug-resistant Gram-negative pathogens in Japan has the potential to save 4 249 096 life years, corresponding to 3 602 311 quality-adjusted life years. The associated maximum clinical and economic gains were estimated at up to 4 422 284 bed days saved, up to 3 645 480 defined daily doses of antibiotics avoided, up to ¥117.6 billion (US 1.1billion)savedinhospitalizationcosts,andanetmonetarybenefitofupto¥18.1trillion(US169.8 billion).

**Discussion:** Learnings from this study can be used by the Japanese government to help inform decision-making on the strategies that may be included in the upcoming National AMR Action Plan and facilitate allocation of the required budget.

**Conclusions:** This analysis demonstrated the considerable economic and clinical value of reducing AMR levels of three Gram-negative pathogens in Japan and could be utilized to support the valuation of antimicrobial treatment and resistance in Japan and more broadly.

## Introduction

Antimicrobial resistance (AMR) represents a significant global public health crisis and, with its increasing prevalence, treatment options available to patients infected with resistant pathogens become increasingly limited. Each year 700 000 people die from AMR-related causes globally, a figure that is projected to rise to 10 million by 2050 at a cost of US $100 trillion dollars to the global gross domestic product if we do not take action.^1^ Although the overall number of deaths attributed to AMR in Japan is not known, a recent study estimated that deaths due to bloodstream infections caused by two pathogens, methicillin-resistant *Staphylococcus aureus* and fluoroquinolone-resistant *Escherichia coli* exceed 8000 per year.^2^ The threat of a rapid rise in resistance rates after the emergence of Coronavirus disease 2019 pandemic is realistic, given that a global spread of both resistant bacteria and AMR-conveying genes are driven by human migration.^3^

Even though AMR occurs naturally, both human interventions and, paradoxically, inaction are further increasing resistance rates and therefore exacerbating the situation. On one hand, any use of antibiotics contributes to the development of AMR, and their overuse and misuse can considerably accelerate it.^4^ Consequently, a growing number of infections are becoming harder to treat as currently available antibiotics become less effective. Increasing AMR means that treatment options for multi-drug resistant bacterial infections are decreasing. Furthermore, the stagnant development of antimicrobial agents is a global problem^1^ and the number of antimicrobial agents developed in Japan is even lower than in the European Union and the United States,^5^ highlighting the need for appropriate antimicrobial stewardship practices (ASPs) in Japan.^5^ Furthermore, despite ample availability of anti-Gram-positive agents, including anti-methicillin-resistant *Staphylococcus aureus* and anti-Vancomycin-resistant *Enterococci* agents, there is a lack of agents against Gram-negative bacteria and carbapenem-resistant *Enterobacterales* remains a threat.

As a result of this global crisis, an international effort is now underway to tackle the threat of AMR. In 2015, the World Health Organization published the Global Action Plan on AMR, calling for a “one health” response.^6^ Consequently, in 2016, the Japanese government produced the National Action Plan on AMR, outlining the measures to slow the emergence of AMR and prevent its spread.^7^ The current AMR National Action Plan spans the 2016–2020 time period and is due to be reviewed and updated. This represents a key opportunity to drive an international response to the AMR epidemic by providing clinical and economic projections on the benefits of reducing AMR.

In this study, we adapted to the Japanese setting a simplified version of a previously published and validated dynamic health economic model of AMR to estimate the clinical and economic consequences of different AMR forecasts, with the aim to guide resource allocation defined within the upcoming National AMR Action Plan.

## METHODS

### Patients and Methods

We adapted a previously developed AMR dynamic cost-effectiveness model^8^ to evaluate the impact of varying drug-resistant Gram-negative pathogen levels on clinical and economic outcomes in Japan. The model utilizes the decision tree treatment pathway component from the previously published and validated dynamic model of AMR^8^ to estimate economic and clinical outcomes (quality-adjusted life years [QALYs] gained, and life years [LY] lost, hospital length of stay [LOS], defined daily dose [DDD] of antibiotics, hospitalization costs, and net monetary benefit [NMB]).

### Patient Population and Setting

Gram-negative infections caused by *Escherichia coli (E. coli)*, *Klebsiella pneumoniae (K. pneumoniae)*, and *Pseudomonas aeruginosa (P. aeruginosa)* include complicated urinary tract infections (cUTIs), complicated intra-abdominal infections (cIAIs), or hospital-associated pneumonia including ventilator-associated pneumonia (HAP/ VAP) were included in the model. These were selected because infections with gram-negative bacteria are highly problematic in terms of resistance development with UTIs, IAIs, and HAP being common among carbapenem-resistant *Enterobacterales* and multi-drug resistant *P. aeruginosa* (MDRP) infections in the Japan Medical Data Vision (MDV).

The annual infected population and corresponding numbers exhibiting resistance in Japan were not directly available and were estimated by using available data from a hospital-based administrative claims database, MDV (with >30 million accumulated patients, across public and private health-care institutes, since April 2003), and the national surveillance program (Japan Nosocomial Infections Surveillance [JANIS]),^9^ and then extrapolating the data to the general population of Japan. MDV data was obtained for patients aged 15 years and older due to differences in the treatment of infectious disease between this patient population and pediatric patients. Three different scenarios were assessed in which different targeted populations available in the MDV database (Table 1) were used to provide estimates of the annual infected population and corresponding resistance levels in Japan:

**Table 1.** Targeted Populations Used To Estimate the Annual Infected Population and Corresponding Resistance Levels in Japan

	**Population A**	**Population B**	**Population C**
**Annual Infections**	722 627	1 473 239	540 262
**Pip/Taz Resistance**	7.75%	8.96%	9.52%
**Meropenem Resistance**	3.81%	5.29%	5.97%
**Diagnosis Breakdown (%)**			
cUTI	59.44	36.44	30.70
cIAI	22.83	31.33	29.64
HAP/VAP	17.73	32.24	39.66
**Pathogen Breakdown (%)**			
*E. coli*	52.36	41.62	36.85
*K. pneumoniae*	25.05	26.09	26.36
*P. aeruginosa*	22.59	32.29	36.79

Abbreviations: cIAI, complicated intra-abdominal infection; cUTI, complicated urinary tract infection; HAP, hospital-acquired pneumonia; Pip/Taz, piperacillin/ tazobactam; VAP, ventilator-associated pneumonia.

Antibiotic resistance rates for population A, B and C are based on the proportion of patients with infectious disease within each population.

**Scenario 1:** Population (A) was obtained by extracting data from the MDV inpatients with a diagnosis of interest (cUTI, cIAI, and HAP/VAP) and multiplying the probability of the three pathogens being implicated in each disease. Probabilities were sourced from published literature^10–12^ and are provided in the **Online Supplementary Material**.

**Scenario 2:** Population (B) was obtained by extracting data from the MDV inpatients with a diagnosis of interest (cUTI, cIAI and HAP/VAP). Eligible patients were prescribed with injectable antimicrobial agents that are covered by National Health Insurance and can be used in current clinical practice with possible antibacterial sensitivity against the three modelled pathogens: *E. coli*, *K. pneumoniae,* and *P. aeruginosa*, including generic products. Of note the antibiotic agent that was prescribed to the most patients was piperacillin/tazobactam (first), followed by meropenem (second).

**Scenario 3:** Population (C) was obtained by extracting from the MDV inpatients with a diagnosis of interest and prescribed piperacillin/tazobactam or meropenem, including generic products.

To note, outpatient costs were not evaluated as Gram-negative bacteria are mostly associated with nosocomial infections.

To estimate the number of patients across Japan who correspond to the three targeted populations, the ratio between the number of new hospitalized patients from the MDV data relative to the number of new hospitalized patients across Japan was calculated for the same period (January to December 2019). Thus, a scaling factor of 6.039 was applied, derived from the number of new admissions in general wards in Japan from the Ministry of Health, Labor and Welfare^13^ and the number of new admissions included in the MDV in 2019.

Antibiotic resistance rates were calculated for each population since resistance rates of the three pathogens differed between populations. Hence, antibiotic resistance rates were based on the proportion of patients with infectious disease for each population.

### Model Structure

**Figure 1.**
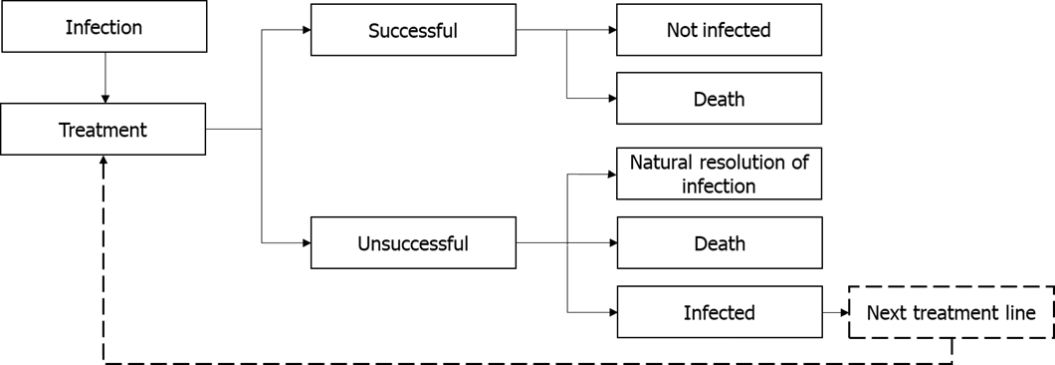
Health Economic Model (treatment pathway) Structure Abbreviations: LOS, length of stay. Infected individuals, denoted by the annual incidence of infection, enter the treatment pathway and are treated according to the pre-determined anti-infective regimen, Piperacillin/Tazobactam followed by Meropenem, until they are either cured (successful treatment/naturally resolves infection) or die (from infection or at a rate aligned to the general population). The LOS associated with a successful treatment is 7 days. In case of unsuccessful treatment, patients may receive up to two treatment lines and a rate of death associated with the modelled indications is applied. Subsequent lines of treatment are received only if a treatment is unsuccessful, and the infection is not resolved naturally. It is assumed that unsuccessful treatment will become apparent before the full course of antibiotic treatment is complete and is associated with a shorter LOS of 4 days. Patients who have exhausted all available antibiotic treatment options and fail to clear the infection naturally are assumed to die from infection 3 days after their last available treatment.

Figure 1 shows the adapted AMR dynamic economic model (treatment pathway), utilized for determining the health impact of the treatment strategy in the context of the modelled infectious environment. Infected patients in the decision tree component receive a specified antibiotic treatment and, as a result, are either cured (successfully treated or infection naturally resolves) or die from infection. Patients who are unsuccessfully treated progress onto the subsequent therapeutic option. Therefore, the modelled treatment pathway comprises a maximum of two lines of treatment. Piperacillin/tazobactam was assumed to be the first-line treatment and meropenem to be the second-line treatment. Selection of antimicrobial treatments was based on the frequently used antimicrobials for Gram-negative bacteria when counted as a generic name from the MDV database. Furthermore, in Japan, the first- and second-line therapies for Gram-negative AMR in clinical practice are piperacillin/tazobactam and meropenem, respectively. Clinical efficacy was assumed to be equivalent for each treatment. The model assumes all patients diagnosed as infected (ie, annual infected population) are treated. In scenarios modelled in populations B and C, the estimated number of annual infections are based on treated populations reducing the impact of this assumption.

### Data Source

Pathogen-specific resistance levels for each treatment were informed by the national surveillance program JANIS.^9^ Indication-specific default hospitalization costs were informed by Japanese diagnosis procedure combinations (DPC) costs; source costs referred to daily hospitalization costs for diagnosis and treatment for each indication.^14^ Daily hospitalization costs were accrued by patients throughout the treatment period (4 days for first-line treatment, and an additional 7 days for second-line treatment). The LOS associated with mortality was assumed to be 3 days, and the cost of death was assumed to be equivalent to the corresponding indication-specific hospitalization cost. Utility values for patients not infected were derived from population norms for a Japanese population aged 70 years and older.^15^ Life expectancy was estimated from the mean age (65–68 years old) of the indication specific infected population, and Japanese life tables (22.13 years at 65–69 years old).^16,17^ To calculate QALYs, the estimated life expectancy was multiplied by the utility values.

Where possible, local model inputs were sourced to better reflect the clinical environment and AMR levels in Japan. Key inputs are summarized in Table 2 and further indication-specific inputs are presented in Table 3.

**Table 2.** Key Inputs Used to Populate the Model

**Model Input**	**Description**	**Local Value**	**Source**
LOS - Successful treatment	The LOS (per-therapy line) of a patient when a line of treatment is successful (days).	7 days	Local expert opinion
LOS - Unsuccessful treatment	The LOS (per-therapy line) of a patient when a line of treatment is unsuccessful (days).	4 days	Local expert opinion
Additional LOS for mortality	An additional LOS associated with patients who die in hospital (days).	3 days	Local expert opinion
Utility (not infected)	Health state utility for patients whose infection has been resolved.	0.8472*	Shiroiwa et al.^15^
Life expectancy post treatment success	The life expectancy of a successfully treated patient based on 65-69-year-olds in Japan.±	22.13 years	National Institute of Population and Social Security Research^17^
Mortality rate (given successful treatment)	The daily rate of mortality associated with successful treatment.	0.0000255	National Institute of Population and Social Security Research^17^
Treatment efficacy (given no resistance)	The probability of treatment success in patients with no resistance to treatment.	0.9	Assumption
Treatment efficacy (given resistance)	The probability of treatment success in patients with resistance to treatment.	0.03	Assumption

Abbreviations: LOS, length of stay.

* Weighted mean (based on sex) of Japanese population norm aged ≥70 years old

± Based on the weighted mean age of the indication specific infected population from UK hospital admission data (65–68 years old) and the diagnosis breakdown in Japan for Population A, B and C (Table 1).^17^

**Table 3.** Additional Indication Specific Inputs

**Input**	**Input Description**	**Input Value**	**Source**
**cUTI Specific Inputs**			
Utility (infected)	The health state utility of an infected cUTI patient.	0.68	Ernst et al.^18^
Mortality rate (given unsuccessful treatment)	The daily rate of mortality associated with a failing treatment on a cUTI patient.	0.002151*	Fukunaga et al.^19^
Daily hospitalization cost	The cost associated with each day a cUTI patient spends in the general ward.	¥28 410 (US $266)	Japan DPC code 110310xx99xx0x as of 2021
**cIAI Specific Inputs**			
Utility (infected)	The health state utility of an infected cIAI patient.	0.6	Brasel et al.^20^
Mortality rate (given unsuccessful treatment)	The daily rate of mortality associated with a failing treatment on a cIAI patient.	0.011812*	Niwa et al.^21^
Daily hospitalization cost	The cost associated with each day a cIAI patient spends in the general ward.	¥30 280 (US $284)	Japan DPC code 060370xx99x0xx as of 2021
**HAP/VAP Specific Inputs**			
Utility (infected)	The health state utility of an infected HAP/VAP patient.	0.58	Beusterien et al.^22^
Mortality rate (given unsuccessful treatment)	The daily rate of mortality associated with a failing treatment on a HAP/VAP patient.	0.012080*	The JRS Guidelines for the Management of Pneumonia in Adults^10^
Daily hospitalization cost	The cost associated with each day a HAP/VAP patient spends in the general ward.	¥28 700 (US $269)	Japan DPC code 0400800399x00x as of 2021

Abbreviations: cIAI, complicated intra-abdominal infection; cUTI, complicated urinary tract infection; DPC, diagnosis procedure combination; HAP, hospital- acquired pneumonia; JRS, Japanese Respiratory Society; VAP, ventilator-associated pneumonia.

*Published literature reporting mortality over 30-days in a Japanese setting and converted to a daily rate. For cUTI, Fukunaga et al,^19^ a study reporting 6.25% deaths among urosepsis patients over 30 days, was used. For cIAI, Niwa et al,32 a study reporting 11.4% deaths associated with bloodstream infections over 30 days and a hazard ratio of 2.92 for IAI, was used. For HAP/VAP, The JRS Guidelines for the Management of Pneumonia in Adults,^10^ a study reporting a 30-day mortality rate of 30.4% for HAP patients, was used.

### Cost-effectiveness Analysis

The cost-effectiveness analysis assessed both clinical and economic outcomes as a function of varying AMR levels of three Gram-negative pathogens in Japan (increasing or decreasing relative to the current level by up to 100%). The outcomes were evaluated for each population (A, B and C) and included hospital LOS (combining the number of days spent in hospital due to treatment and LOS due to mortality) and the corresponding cost of hospitalization based on indication-specific general ward costs, DDD, the number of LYs lost as a result of infection (LYs lost), and the number of QALYs lost as a result of infection (QALYs lost). For each population, current AMR levels of three Gram-negative pathogens were increased/reduced in order to evaluate the impact on incremental benefits. The model was run over a 10-year time horizon with a willingness-to-pay threshold of ¥5 000 000 (US $46 827), in line with the most conservative threshold used in the Japan Health Technology Assessment to estimate NMB.^23^ Both costs and benefits were discounted at a rate of 2% per annum in accordance with health technology assessment guidance in Japan.^24^ The Japanese Yen was converted to US dollars using the average rate in 2020 (US $1=¥106.775) published by the Organization for Economic Cooperation and Development.^25^

### Sensitivity Analysis

One-way sensitivity analyses (OWSA) were conducted on key model input parameters, listed in Table 2. Each input was adjusted by ±20%, with the impact on hospitalization costs saved and QALYs gained assessed in population A.

## RESULTS

### Population-based Estimates of Value Associated with Gram-negative AMR Reduction

The absolute and incremental outcomes derived based on populations A-C are presented in Tables 4-6. Extrapolating population B to the general Japanese population size was associated with the greatest costs, resource use, and LY and QALY loss. This is consistent with the largest annual number of infections among the 3 populations included in the model. Using population B, a 50% reduction in AMR levels of three Gram-negative pathogens was estimated to save 2 328 109 LYs, corresponding to 1 973 612 QALYs, and freeing up 2 210 023 hospital bed days over 10 years, generating a savings of ¥58.8 billion (US $550.5 million) in hospitalization costs (Table 5).

**Table 4.** Absolute and Incremental (relative to current resistance level) Outcomes Using Population A

**Model Outcome**		**1-year Outcomes**			**10-year Outcomes**		
		**Current Resistance**	**50% Reduction in Resistance**	**50% Increase in Resistance**	**Current Resistance**	**50% Reduction in Resistance**	**50% Increase in Resistance**
Hospital LOS (days)	Absolute	5 529 661	5 434 982	5 624 270	55 296 610	54 349 823	56 242 700
	Incremental	–	- 94 679	94 609	–	- 946 787	946 090
Defined daily dose	Absolute	5 474 753	5 395 603	5 551 539	54 747 534	53 956 031	55 515 395
	Incremental	–	- 79 150	76 786	–	- 791 502	767 861
Hospitalization costs (¥, USD)	Absolute	¥159 737 337 604 (US $1 496 018 147)	¥157 003 266 968 (US $1 470 412 240)	¥162 469 397 348 (US $1 521 605 220)	¥1461 015 140 157 (US $13 683 120 020)	¥1436 008 346 793 (US $13 448 919 193)	¥1486 003 541 180 (US $13 917 148 595)
	Incremental	–	-¥2 734 070 636 (US -$25 605 906)	¥2 732 059 744 (US $25 587 073)	–	-¥25 006 793 364 (US -$234 200 828)	¥24 988 401 024 (US $234 028 574)
Life years lost*	Absolute	328 374	235 507	434 964	3 003 430	2 154 028	3 978 338
	Incremental	–	- 92 868	106 590	–	- 849 402	974 909
QALYs lost+	Absolute	281 274	202 544	371 629	2 572 632	1 852 539	3 399 054
	Incremental	–	- 78 730	90 355	–	- 720 093	826 422

Abbreviations: LOS, length of stay; QALY, quality-adjusted life-year.

*Life years lost based on a life expectancy of 22.1 years after treatment prior to discounting.

+QALYs lost based on a quality adjusted life expectancy of 18.7 years after treatment prior to discounting.

**Table 5.** Absolute and Incremental (relative to current resistance level) Outcomes Using Population B

							
**Model Outcome**		**1-year Outcomes**			**10-year Outcomes**		
		**Current Resistance**	**50% Reduction in Resistance**	**50% Increase in Resistance**	**Current Resistance**	**50% Reduction in Resistance**	**50% Increase in Resistance**
Hospital LOS (days)	Absolute	11 324 310	11 103 308	11 545 088	113 243 101	111 033 078	115 450 885
	Incremental	–	- 221 002	220 778	–	-2 210 023	2 207 784
Defined daily dose	Absolute	11 189 782	11 011 342	11 360 556	111 897 822	110 113 416	113 605 562
	Incremental	–	- 178 441	170 774	–	-1 784 406	1 707 739
Hospitalization costs (¥, USD)	Absolute	¥329 406 466 854 (US $3 085 052 370)	¥322 979 769 416 (US $3 024 863 212)	¥335 826 656 376 (US $3 145 180 579)	¥3 012 870 018 733 (US $28 216 998 536)	¥2 954 089 132 565 (US $27 666 486 842)	¥3 071 591 381 157 (US $28 766 952 762)
	Incremental	–	-¥6 426 697 438 (US -$ 60 189 159)	¥6 420 189 522 (US $60 128 209)	–	-¥58 780 886 168 (US -$550 511 694)	¥58 721 362 424 (US $549 954 225)
Life years lost*	Absolute	804 542	550 003	1 103 593	7 358 631	5 030 522	10 093 862
	Incremental	–	- 254 539	299 051	–	-2 328 109	2 735 230
QALYs lost+	Absolute	688 566	472 785	942 058	6 297 873	4 324 262	8 616 397
	Incremental	–	- 215 781	253 491	–	-1 973 612	2 318 524

Abbreviations: LOS, length of stay; QALY, quality-adjusted life-year.

*Life years lost based on a life expectancy of 22.1 years after treatment prior to discounting.

+QALYs lost based on a quality adjusted life expectancy of 18.7 years after treatment prior to discounting.

**Table 6.** Absolute and Incremental (relative to current resistance level) Outcomes Using Population C

**Model Outcome**		**1-year Outcomes**			**10-year Outcomes**			
		**Current Resistance**	**50% Reduction in Resistance**	**50% Increase in Resistance**	**Current Resistance**	**50% Reduction in Resistance**	**50% Increase in Resistance**	
Hospital LOS (days)	Absolute	4 162 035	4 076 148	4 247 825	41 620 353	40 761 476	42 478 250	
	Incremental	–	- 85 888	85 790	–	- 858 877	857 897	
Defined daily dose	Absolute	4 109 142	4 040 745	4 174 176	41 091 422	40 407 452	41 741 758	
	Incremental	–	- 68 397	65 034	–	- 683 970	650 336	
Hospitalization costs (¥, USD)	Absolute	¥121 025 895 572 (US $1 133 466 594)	¥118 529 020 062 (US $1 110 082 136)	¥123 519 923 396 (US $1 156 824 382)	¥1 106 946 368 542 (US $10 367 093 126)	¥1 084 109 047 117 (US $10 153 210 462)	¥1 129 757 644 014 (US $10 580 731 857)	
	Incremental	–	-¥2 496 875 509 (US -$23 384 458)	¥2 494 027 824 (US $23 357 788)	–	-¥22 837 321 425 (US $- 213 882 664)	¥22 811 275 472 (US $213 638 731)	
Life years lost*	Absolute	316 327	211 724	440 459	2 893 238	1 936 500	4 028 593	
	Incremental	–	- 104 603	124 132	–	- 956 738	1 135 355	
QALYs lost+	Absolute	270 619	181 945	375 837	2 475 175	1 664 132	3 437 542	
	Incremental	–	- 88 674	105 219	–	- 811 043	962 366	

Abbreviations: LOS, length of stay; QALY, quality-adjusted life-year.

\*Life years lost based on a life expectancy of 22.1 years after treatment prior to discounting.
          +QALYs lost based on a quality adjusted life expectancy of 18.7 years after treatment prior to discounting.

Estimates based on populations A (Table 4) and C (Table 6) were substantially smaller than for population B and relatively similar to each other, with higher economic gains realized by reducing Gram-negative AMR in population A than C and the converse observed for LYs and QALYs.

### The Relationship Between Reduction in Gram-negative AMR and Clinical and Economic Outcomes

**Figure 2.**
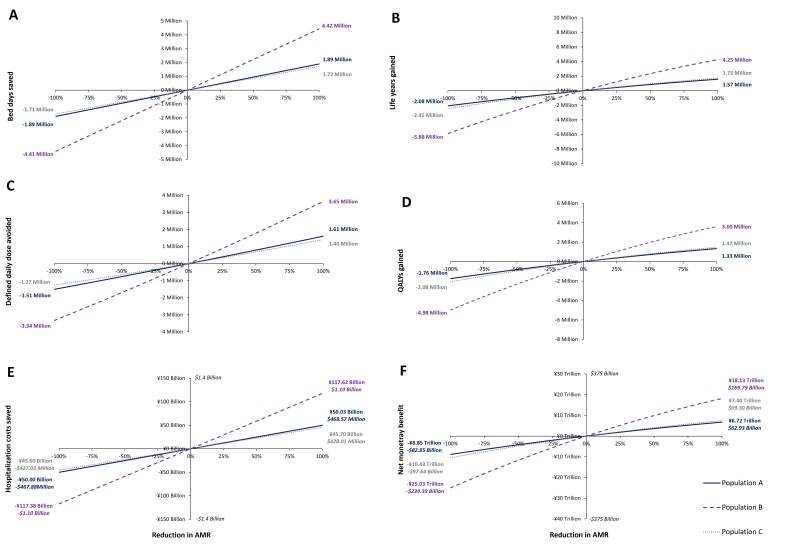
Effects of Varying AMR Levels on Clinical and Economic Outcomes The effect of alternative levels of AMR on outcomes of interest is presented in Figure 2. The horizontal axis represents percentage reduction in AMR levels from 100% of the current level (corresponding to no resistance) to -100% of the current level (corresponding to doubled resistance incidence). Based on the number of infections per year over a 10-year period, reducing AMR in Japan has the potential to save 4 249 096 life years, corresponding to 3 602 311 QALYs. The maximum economic gains realized over a 10-year period from reducing AMR levels were estimated at up to 4 422 284 bed days saved, 3 645 480 DDDs avoided, ¥117.6billion saved in hospitalization costs and a NMB of up to ¥18.1 trillion.

Figure 2 presents the effect of varying AMR levels of three Gram-negative pathogens on outcomes of interest. The horizontal axis represents the percentage reduction in AMR levels of three Gram-negative pathogens from 100% of the current level (corresponding to no resistance) to -100% of the current level (corresponding to doubled resistance incidence). Based on the number of infections per year over a 10-year period, reducing AMR levels of three Gram-negative pathogens in Japan has the potential to save 4 249 096 life years, corresponding to 3 602 311 QALYs.

The maximum economic gains realized over a 10-year period from AMR levels of three Gram-negative pathogens were estimated at up to 4 422 284 bed days saved, up to 3 645 480 DDDs avoided, up to ¥117.6 billion (US $1.1 billion) saved in hospitalization costs, and an NMB of up to ¥18.1 trillion (US $169.8 billion).

### One-way Sensitivity Analysis

**Figure 3.**
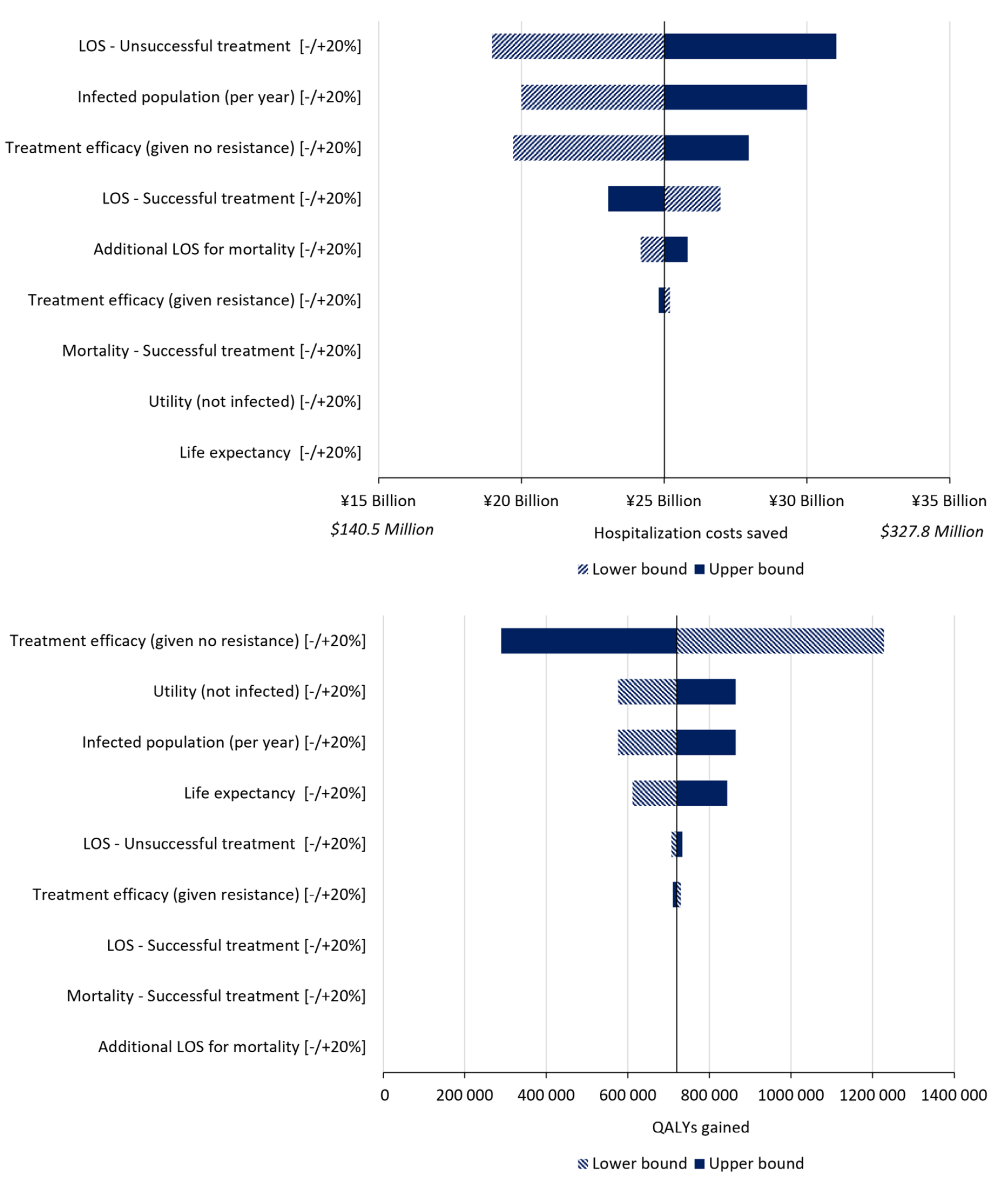
One Way Sensitivity Analysis (OWSA) Varying Key Inputs by +/-20% - Population A A OWSA varying key inputs by ±20% was conducted for incremental outcomes of hospitalization costs and QALYs lost for resistance levels reduced by 50% against current resistance levels in Population A. Treatment efficacy (given no resistance) and the infected population size (per year) were key drivers of both clinical and economic outcomes. Furthermore, LOS was a key driver of clinical outcomes and utility (not infected) and life expectancy were key drivers of economic outcomes.

Varying the input for treatment efficacy (given no resistance) had the greatest impact on QALYs gained. Estimates for LOS for unsuccessful treatment had the largest impact on hospitalization costs saved. Hospitalization costs saved were also sensitive to estimates for infected population (per year) and treatment efficacy (given no resistance). Figure 3 displays the impact of all assessed input parameters in a tornado plot.

## DISCUSSION

The analysis undertaken in this study demonstrates the potential clinical and economic savings from reducing future Gram-negative AMR levels in Japan, as well as the threats associated with potential increases in Gram-negative AMR levels. JANIS-derived populations A, B, and C represent different scenarios in which a reduction in Gram-negative AMR may be realized, with population A focused on specific pathogens (*E. coli, K. pneumoniae,* and *P. aeruginosa*) causing cUTI, cIAI, and HAP/VAP, and population C on cUTI, cIAI, and HAP/VAP treated with piperacillin/tazobactam or meropenem. Population B was a broad population of patients with cUTI, cIAI, and HAP/VAP treated with agents potentially effective against *E. coli, K. pneumoniae,* and *P. aeruginosa*. The broadest population (B), having the highest annual number of infections, gained the greatest clinical and economic benefits from AMR reduction. The outcomes estimated for populations A and C were broadly similar, with a larger potential resource and cost savings attained in population A, owing to the higher annual number of infections than in population C. Conversely, LYs and QALYs gained were greater in population C, in which baseline resistance was higher than in population A. Patients whose infections are resistant to both piperacillin/tazobactam and meropenem have potentially very limited treatment options, so that a reduction in AMR in this setting was associated with high LY and QALY gains.

The NMB of ¥18.1 trillion (US $169.8 billion) represents the maximum value of investment according to the Japanese willingness-to-pay threshold and can be used by the Japanese government to help inform decision-making on the strategies that may be included in the upcoming National AMR Action Plan and facilitate allocation of the required budget. This figure, however, is likely to be an underestimation since our study focuses only on a limited number of indications and pathogens. Therefore, when considering other infections with high prevalence, such as methicillin-resistant *Staphylococcus aureus*, the actual economic savings will be greater. Nevertheless, the results of this study provide a clear motivation for incentivizing global research and development initiatives, in order to achieve the benefits associated with lowering AMR levels.

Reducing the use of inappropriate antimicrobial agents is one way of lowering AMR rates, as evidenced in a study by Terahara et al, which showed an association between fluoroquinolone consumption and *E. coli.* resistance on a national scale in Japan.^26^ Other evidence has demonstrated a positive correlation between carbapenem use and the proportion of carbapenem-resistant *P. aeruginosa* isolates.^27^ In addition, interventions to reduce AMR already exist, and are proving to be successful. Such interventions include ASPs that may limit the use of specific antibiotics, regulate treatment duration, and incorporate auditing.^28,29^ In Japan, a sustained ASP implementation with additional consultation was shown to reduce AMR in a pediatric population.^30^ Similarly, another study in Spain evaluated the impact of ASP implementation on meropenem prescription and subsequent effect on clinical and economic outcomes.^31^ This study found that lowering meropenem usage through ASP implementation reduced costs and mortality associated with hospital-acquired multidrug-resistant bloodstream infection.

Morel et al presented a framework guiding AMR-related cost assessments across different localizations, with the aim that this can feed into both more detailed analyses comparing AMR-related interventions at the local level and broader analyses aiming to capture the economic burden of AMR at a global level.^32^ In our analysis, extrapolations were made using Japanese local data. While caution should be used in attempting to generalize the analysis presented in our study to other countries, even within the Asia-Pacific region, the message that economic and clinical benefits can be achieved by reducing AMR can be applied globally. The need for an international strategy that includes antimicrobial use in human and animal health, and its environmental impact, is evident.

While efforts to optimize the use of existing antimicrobials are ongoing, there is stagnation in the development of novel antimicrobial agents, owing to the lack of investment in this area. This is a particular problem in Japan, where only 6 antibiotic agents have been approved since 2002. Conversely, within the same time-frame, the amount of antibiotic agents approved in the United States and the European Union has almost quadrupled this amount,^5^ an indication that the development situation of antimicrobial agents in Japan is not as great as in the United States and the European Union.

Whilst the development of antimicrobial agents has halted in recent years, efforts are being made in some countries to overcome this issue, such as in the United Kingdom, the United States and Sweden. In the United Kingdom, an antimicrobial reimbursement pilot program has been launched,^33^ a scheme that rewards the manufacturer of an antimicrobial agent based on the medicine’s value to the health-care system rather than the volume of drug used. Whilst this and similar efforts encourage pharmaceutical companies to kick-start the development of novel antimicrobial agents, the appropriate use of new and existing antimicrobial agents will remain essential to limit the increase in AMR.

There are some limitations to this study that need to be addressed. First, although this analysis may help inform the value of reducing AMR levels of three Gram-negative pathogens, or the burden associated with their increase, it does not consider the methods required to achieve these outcomes, including any additional costs they might incur. Second, changes in future populations, including demographic changes such as the growth in the number of older people, were not considered. Third, the number of pathogens evaluated was limited to 3 due to the constraints of the model structure. Fourth, the diseases and pathogens analyzed were limited. According to the data published by JANIS, the isolation rates of the three organisms (*E. coli, K. pneumoniae*, and *P. aeruginosa*) were 13.88%, 6.23%, and 6.56%, respectively, of the total number of patients with available specimens. While the pathogens considered are frequently implicated in nosocomial infection, the generalizability of our results to other bacterial species is uncertain. Fifth, in Japan, hospitalization costs are calculated on a DPC basis in DPC hospitals (mostly acute hospitals) and on a fee-for-service reimbursement basis in non-DPC hospitals (mostly small- and middle-sized non-acute hospitals). Nevertheless, the DPC costs were used in the results. Sixth, the model does not account for the additional benefits of reduced AMR, including reduced secondary transmission, the impact of infection on pregnant women, or prophylactic use of antimicrobial agents in surgery. Seventh, hospitalization costs have not been adjusted for inflation over the 10 years assessed and are therefore likely underestimated. Finally, estimates of hospital LOS are uncertain due to the reliance on expert opinion in the absence of empirical research.

## CONCLUSION

Our study shows the economic and clinical value of reducing AMR levels of three Gram-negative pathogens in Japan. With increased efforts now being made globally to reduce AMR rates, our model could be utilized to help support the valuation of antimicrobial treatment and resistance. Successfully updating, implementing, and executing a National AMR Action Plan is necessary to respond robustly to the threat posed by an increase in AMR prevalence.

### Author Contributions

JG, PME, AT, and AY conceptualized and designed the study; RM and OD developed the model under supervision from JG and PME; TO and AY provided local data to inform the model; TM ensured applicability of the model and analysis to the Japanese clinical setting and provided expert guidance to that end. All authors contributed to interpretation of the results, preparation and review of the manuscript, and approval of the final manuscript for publication.

### Conflicts of Interest

The authors declare the following potential conflicts of interest with respect to the research, authorship, and/or publication of this article: TM has been on the speakers’ bureau for Pfizer Japan Inc. and MSD K.K. JG, PM, and RM are employees of Health Economics and Outcomes Research Ltd., which received funding from Pfizer Inc. to undertake the research outlined in this study. OD was an employee of Health Economics and Outcomes Research Ltd. at the time this study was conducted and is now an employee of Public Health Wales. AT is a full-time employee of Pfizer R&D UK Limited. AT and AY hold stocks and stock options from Pfizer Inc. TO and AY are full-time employees of Pfizer Japan Inc.

### Data Availability Statement

The anonymized patient data underlying this manuscript are derived from the Medical Data Vision database and the national surveillance program (Japan Nosocomial Infections Surveillance) and cannot be made available by the authors.

## Supplementary Material

Online Supplementary Material
