# Supplementary material for: Estimating the Economic and Clinical Value of Reducing Antimicrobial Resistance to Three Gram-negative Pathogens in Japan
Source: J Health Econ Outcomes Res. 2021 Oct 6;8(2):64–75. doi: 10.36469/001c.28327 (PMC8494726; doi:10.36469/001c.28327)
Supplement: Online Supplementary Material [file jheor_2021_8_2_28327_72939.pdf]

Journal of Health Economics and Outcomes Research

Online Supplementary Material

Matsumoto T, Darlington O, Miller R, et al. Estimating the Economic and Clinical Value of Reducing Antimicrobial Resistance to Three Gram-negative Pathogens in Japan. *JHEOR*. 2021;8(2)64-75. doi:10.36469/jheor.2021.28327

Table S1. Proportion of Pathogens for Each Indication
Table S2. Calculation of the Patient Numbers Within Each Scenario by Pathogen Using MDV
Table S3. Calculation of Resistance Rates for PIP/TAZ and MEMP for Each Scenario (pooled by pathogen)
References

This supplementary material has been provided by the authors to give readers additional information about their work.

This is an open-access article distributed under the terms of the Creative Commons Attribution 4.0 International License (CCBY-4.0). View this license's legal deed at http://creativecommons.org/licenses/by/4.0 and legal code at http://creativecommons.org/licenses/by/4.0/legalcode for more information.

Table S1. Proportion of Pathogens for Each Indication

Input	Input Value	Source
HAP		
Proportion *E.coli*	0.00%	The JRS Guidelines for the Management of Pneumonia in Adults[1]
Proportion *Klebsiella pneumoniae*	5.40%	
Proportion *P.aeruginosa*	13.90%	
**Total**	**19.30%**	NA
VAP		
Proportion *E.coli*	2.50%	The JRS Guidelines for the Management of Pneumonia in Adults[1]
Proportion *Klebsiella pneumoniae*	6.40%	
Proportion *P.aeruginosa*	34.80%	
**Total**	**43.70%**	NA
UTI		
Proportion *E.coli*	39.84%	Kobayashi et al.[2]
Proportion *Klebsiella pneumoniae*	13.84%	
Proportion *P.aeruginosa*	5.79%	
**Total**	**59.47%**	NA
IAI		
Proportion *E.coli*	17.50%	Mikamo et al.[3]
Proportion *Klebsiella pneumoniae*	8.75%	
Proportion *P.aeruginosa*	5.62%	
**Total**	**31.87%**	NA

Abbreviations: HAP, hospital-acquired pneumonia; IAI, intra-abdominal infection; JRS, Japanese Respiratory Society; UTI, urinary tract infection; VAP, ventilator-associated pneumonia.

Table S2. Calculation of the Patient Numbers Within Each Scenario by Pathogen Using MDV

Input	Input Value			Source/calculation
	E.coli	*Klebsiella pneumoniae*	*P.aeruginosa*	
**Pathogen Proportion Within Each Indication**				
Pathogen proportion for HAP; %	0.00%	27.98%	72.02%	Scaled based on the pathogen distribution shown in Table S1
Pathogen proportion for VAP; %	5.72%	14.65%	79.63%	
Pathogen proportion for UTI; %	66.99%	23.27%	9.74%	
Pathogen proportion for IAI; %	54.91%	27.46%	17.63%	
**Scenario A - Patient Numbers**				
Total HAP patients; n		109 307		MDV; Unique number of in-patients, 15 years or older and diagnosis (HAP, VAP, UTI, and IAI) ‡
Total VAP patients; n		271		
Total UTI patients; n		119 601		
Total IAI patients; n		85 708		
**Total; n (%)**	**62 655 (52.36%)**	**29 972 (25.05%)**	**27 030 (22.59%)**	Calculation; Sum of pathogen distribution (Table S1) multiplied by patient numbers
**Scenario B - Patient Numbers**				
Total HAP patients; n		78 416		MDV; Unique number of in-patient with 15 years or older with diagnosis (HAP, VAP, UTI, and IAI) and prescribed possible antimicrobial agents with antibacterial sensitivity against 3 species‡
Total VAP patients; n		222		
Total UTI patients; n		88 890		
Total IAI patients; n		76 420		
**Total; n (%)**	**101 524 (41.62%)**	**63 641 (26.09%)**	**78 783 (32.26%)**	Calculation; Sum of pathogen proportion within each indication multiplied by patient numbers
**Scenario C - Patient Numbers**				
Total HAP patients; n		35 342		MDV; Unique number of in-patient with 15 years or older with diagnosis (HAP, VAP, UTI, and IAI) and prescribed PIP/TAZ or MEMP‡
Total VAP patients; n		142		
Total UTI patients; n		27 462		
Total IAI patients; n		26 514		
**Total; n (%)**	**32 964 (36.85%)**	**23 580 (26.36%)**	**32 916 (36.79%)**	Calculation; Sum of pathogen proportion within each indication multiplied by patient numbers

Abbreviations: MDV, Medical Data Vision; HAP, hospital-acquired pneumonia; IAI, intra-abdominal infection; MEMP, Meropenem; PIP/TAZ, Piperacillin/Tazobactam; UTI, urinary tract infection; VAP, ventilator-associated pneumonia.

The count results of hospital acquired pneumonia may include community-acquired pneumonia patients.
‡The column of "Total" is not unique number of patients. It is the total of 4 unique patient numbers.

Table S3. Calculation of Resistance Rates for PIP/TAZ and MEMP for Each Scenario (pooled by pathogen)

Input	Input Value			Source/calculation
	E.coli	*Klebsiella pneumoniae*	*P.aeruginosa*	
**Resistance Rates by Pathogen**				
PIP/TAZ resistance	4.90%	5.10%	17.30%	JANIS[4]
MEMP resistance	0.30%	0.60%	15.50%	
**Scenario A**				
Proportion of patients	52.36%	25.05%	22.59%	See Table S2
PIP/TAZ resistance (weighted by proportion of patients)	2.57%	1.28%	3.91%	Calculation; Proportion of patients multiplied by PIP/TAZ resistance
MEMP resistance (weighted by proportion of patients)	0.16%	0.15%	3.50%	Calculation; Proportion of patients multiplied by MEMP resistance
**PIP/TAZ Resistance (pooled)**		**7.75%**		Calculation; Sum of PIP/TAZ resistance (weighted by proportion of patients) by pathogen
**MEMP Resistance (pooled)**		**3.81%**		Calculation; Sum of MEMP resistance (weighted by proportion of patients) by pathogen
**Scenario B**				
Proportion of patients	41.62%	26.09%	32.29%	See Table S2
PIP/TAZ resistance (weighted by proportion of patients)	2.04%	1.33%	5.59%	Calculation; Proportion of patients multiplied by PIP/TAZ resistance
MEMP resistance (weighted by proportion of patients)	0.12%	0.16%	5.01%	Calculation; Proportion of patients multiplied by MEMP resistance
**PIP/TAZ Resistance (pooled)**		**8.96%**		Calculation; Sum of PIP/TAZ resistance (weighted by proportion of patients) by pathogen
**MEMP Resistance (pooled)**		**5.29%**		Calculation; Sum of MEMP resistance (weighted by proportion of patients) by pathogen
**Scenario C**				
Proportion of patients	36.85%	26.36%	36.79%	See Table S2
PIP/TAZ resistance (weighted by proportion of patients)	1.81%	1.34%	6.37%	Calculation; Proportion of patients multiplied by PIP/TAZ resistance
MEMP resistance (weighted by proportion of patients)	0.11%	0.16%	5.70%	Calculation; Proportion of patients multiplied by MEMP resistance
**PIP/TAZ Resistance (pooled)**		**9.52%**		Calculation; Sum of PIP/TAZ resistance (weighted by proportion of patients) by pathogen
**MEMP Resistance (pooled)**		**5.97%**		Calculation; Sum of MEMP resistance (weighted by proportion of patients) by pathogen

Abbreviations: MEMP, Meropenem; PIP/TAZ, Piperacillin/Tazobactam.

REFERENCES

1. Japanese Respiratory Society. Adult Pneumonia Clinical Practice Guidelines 2017. https://www.jrs.or.jp/modules/guidelines/index.php?content_id=94. Accessed January 28, 2021.

2. Kobayashi K, Yamamoto S, Takahashi S, et al. The third national Japanese antimicrobial susceptibility pattern surveillance program: bacterial isolates from complicated urinary tract infection patients. *J Infect Chemother*. 2020;26(5):418-428. doi:10.1016/j.jiac.2020.01.004

3. Mikamo H, Takesue Y, Kusachi S, Kotaka M, Kawachi Y, Aikawa N. An open-label multicenter study of tazobactam/piperacillin in the treatment of patients with intra-abdominal infections. *Jpn J Chemother*. 2012;60:560-572.

4. Japan Nosocomial Infections Surveillance (JANIS) Clinical Laboratory Division. Annual Open Report 2019 (All Facilities) https://janis.mhlw.go.jp/english/report/open_report/2019/3/1/ken_Open_Report_Eng_201900_clsi2012.pdf. Published July 3, 2020. Accessed January 18, 2021.
